# Characterization of a new lytic bacteriophage vB_RanS_GDF21 and its endolysin LysGDF21 with antimicrobial activity against *Riemerella anatipestifer*

**DOI:** 10.3389/fmicb.2025.1715949

**Published:** 2026-01-13

**Authors:** Yaru Zhao, Yulan Liu, Yina Jiang, Xiaoting Li, Zhenshu Si, Jianbiao Lu, Shengliang Cao, Xijuan Xue, Yubao Li, Cheng Liu

**Affiliations:** 1 College of Agriculture and Biology, Liaocheng University, Liaocheng, Shandong, China; 2 Phage Research Center, Liaocheng University, Liaocheng, Shandong, China; 3 Shandong Sinder Technology Co., Ltd., Qingdao, China; 4 School of Pharmaceutical Sciences and Food Engineering, Liaocheng University, Liaocheng, Shandong, China

**Keywords:** *Riemerella anatipestifer*, phage vB_RanS_GDF21, endolysin, biofilm, antimicrobial activity

## Abstract

**Introduction:**

*Riemerella anatipestifer* (*R. anatipestifer*) presents as a major pathogen causing septicemia among poultry. The complex serotype diversity and multidrug resistance of *R. anatipestifer* severely compromise infection prevention and treatment strategies. Bacteriophages and their endolysins represent a promising alternative strategy against *R. anatipestifer*.

**Methods:**

The phage vB_RanS_GDF21 (GDF21) was isolated from feces of a duck farm, and its biological properties were characterized. Whole-genome sequencing was performed using the Illumina NovaSeq 6000 platform, followed by a bioinformatic analysis of the genome. The derived endolysin, LysGDF21 (encoded by *orf 65*), was expressed in *Escherichia coli* BL21 (DE3), and its anti-biofilm and antimicrobial activities were assessed.

**Results:**

Transmission electron microscopy showed that phage GDF21 had an icosahedral symmetric head, and a long, non-contractile tail. It exhibited a 20 min eclipse period, burst size of 74 PFU/infected cell, and stability at pH 5-11 and temperatures below 50 °C. Whole-genome sequencing revealed that GDF21 possesses a double-stranded DNA genome with length of 46,925 bp and G+C content of 35.25%. Among the 75 predicted ORFs, 27 were functionally annotated and no genes associated with virulence or antibiotic resistance were identified. Phylogeny and classification analysis indicated that GDF21 is a member of the class Caudoviricetes and is closely related to Riemerella phage vB_RanS_CRP19. LysGDF21 significantly inhibited biofilm formation and disrupted mature biofilms in *R. anatipestifer*. When combined with EDTA, LysGDF21 exhibited broad antibacterial activity against *R. anatipestifer*.

**Conclusion:**

The characterization of phage GDF21 enriches the phage database, while the potent anti-biofilm and antibacterial activities of LysGDF21 highlight its therapeutic potential against *R. anatipestifer* infections.

## 1 Introduction

*Riemerella anatipestifer* (*R. anatipestifer*) infection, commonly referred to as duck infectious serositis, is a serious and highly contagious septic disease caused by *R. anatipestifer*, which primarily targets ducks, geese, turkeys, and various other avian species (Leavitt and Ayroud, 1997; Liang et al., 2024). Currently, it has had a profound impact on the global poultry industry, resulting in substantial economic losses. *R. anatipestifer* primarily infects waterfowl, particularly ducklings, leading to considerable morbidity and mortality (Chang et al., 2019; Lyu et al., 2023). Recent studies have shown an increasing infection of *R. anatipestifer* among other domestic poultry including broilers, laying hens, and breeder hens (Omaleki et al., 2021; Chen Y. et al., 2024). To date, at least 2l serotypes of *R. anatipestifer* have been reported, but no significant cross-protection among these serotypes posing challenges for the development of vaccines against this pathogen (Pathanasophon et al., 2002; Kang et al., 2018; Zheng et al., 2023). Antibiotics have also been utilized to manage *R. anatipestifer* infection in ducks (Zhang et al., 2025). Nevertheless, the long-term, excessive, and unscientific use of antibiotics has resulted in the emergence of multidrug-resistant strains (Zhu et al., 2023; Dong et al., 2024; Liu et al., 2024). The resistance of *R. anatipestifer* to currently available antibiotics has increased significantly, and antibiotic residues have been found in products derived from ducks (Quan et al., 2023; Wang T. et al., 2022). These pose significant threats to both food safety and public health. Therefore, there is an urgent necessity to explore alternative antibacterial strategies aimed at preventing and controlling *R. anatipestifer* infection, where the applications based on bacteriophages (phages) and their endolysins are the most promising candidates.

Phages are viruses that specifically infect bacteria (Dion et al., 2020; Strathdee et al., 2023). With an estimated abundance of 10^31^ across the globe, phages may actually outnumber bacteria by around 10 times (Dion et al., 2020). Phage therapy, a method that has been employed for over a century, has historically been utilized as a treatment for bacterial infections (Cisek et al., 2017; Strathdee et al., 2023). In light of the rise of multidrug-resistant bacterial infections, phage therapy could serve as a promising alternative to current antimicrobial therapies (Kortright et al., 2019; Eskenazi et al., 2022). This potential also extends to agriculture, where phage therapy is used in poultry farming to combat pathogens like *Salmonella* and *E. coli* (Żbikowska et al., 2020; Lorenzo-Rebenaque et al., 2021), providing a sustainable strategy for improving animal health and food safety. *R. anatipestifer* phage can be isolated from various sources such as farm sewage, feces, drinking water and other environments. However, the current database of *R. anatipestifer* phages is quite limited. Only a few *R. anatipestifer* phages have been studied (Cheng et al., 2012; Wang Y. et al., 2022). Furthermore, endolysin is a phage-encoded enzyme that destroys the integrity of bacterial cell walls by hydrolyzing peptidoglycan (Young, 2014; Abdelrahman et al., 2021). However, during the lytic cycle, it requires holin proteins to traverse the inner membrane to reach its target and facilitate cell lysis from within (Young, 2014; Abdelrahman et al., 2021). In contrast, when applied exogenously as purified proteins, they can directly access and hydrolyze the peptidoglycan of bacteria (Gram-negative bacteria require penetration of the outer membrane) (Abdelrahman et al., 2021; Gerstmans et al., 2016). Endolysins often exhibit a broader lytic spectrum compared to their original phage, which makes them great application for control of bacteria (Schuch et al., 2002; Li et al., 2021; Wang F. et al., 2022). Therefore, identification of new phages and their associated endolysin are of great interests as effective strategies for the prevention and control of *R. anatipestifer* infections.

Given the urgent need for alternative strategies to control *R. anatipestifer* infections, we investigated bacteriophages and their endolysins as particularly promising candidates. In the current research, we isolated a new lytic phage, vB_RanS_GDF21 (GDF21), targeting *R. anatipestifer* from the duck feces. We conducted a thorough characterization of its biological properties, genomic traits, and phylogenetic relationships. To better understand the function of its endolysin LysGDF21, we further determined the antibacterial and anti-biofilm activity of LysGDF21. This study expanded the phage database of *R. anatipestifer* and provided a promising alternative treatment for *R. anatipestifer* infections.

## 2 Materials and methods

### 2.1 Bacterial strains, plasmids, and growth conditions

A total of 51 *R. anatipestifer* strains previously obtained were used in this study (Zhu et al., 2023). All of these strains were cultured at 37 °C in tryptone soy broth (TSB) containing 10% newborn calf serum (NBCS). These strains were chosen to assess the phage host range and endolysin antibacterial/anti-biofilm activity analyzed. The vector pET-28a(+) was used for cloning and expression of LysGDF21 in *E. coli* BL21 (DE3).

### 2.2 Isolation and purification of phage

The *R. anatipestifer* R-21 strain served as the host cell for the isolation of phages. A total of 36 duck feces samples were obtained from a duck farm in Guangdong, China. The process of phage isolation and purification followed previously established protocols with minor modifications (Cao et al., 2022). Briefly, the samples were blended with an equal volume of PBS, followed by centrifugation, and the supernatant was then filtered using a sterile 0.22 μm filter (Millipore, United States). After filtration, the filtrate was mixed with *R. anatipestifer* R-21 cells that had been cultivated to their logarithmic growth phase. Phage released from the host bacteria were collected by centrifugation at 10,000 × *g* and filtration through a 0.22 μm filter. Phages were subsequently purified using the double-layer agar plate method (Ren et al., 2025). A 10-fold dilution series of the phage filtrate (10^–1^–10^–8^) was prepared in SM buffer [100 mM NaCl, 10 mM MgSO_4_, 50 mM Tris-Cl (pH 7.5)] and then mixed with *R. anatipestifer* R-21. The mixture was added to 5 mL TSB with 0.75% agar, then poured onto a tryptone soya agar (TSA) plate, and incubated overnight at 37 °C in 5% CO_2_ to form phage plaques. The center of an individual plaque was carefully picked using a sterile micropipette tip and resuspended in separate tubes containing 500 μL SM buffer, centrifuged and filtered as above. This procedure was repeated until the plaques exhibited uniformity in size. The purified phages were preserved at −80 °C in 30% glycerol until use.

### 2.3 Transmission electron microscopy (TEM)

Transmission electron microscopy was employed to observe the morphology of phage GDF21 in detail. Briefly, the purified phage with a concentration of 10^10^ PFU/mL was carefully placed onto a carbon-coated copper grid and subjected to negative staining using 2% phosphotungstic acid for 5 min. The grid was allowed to air dry, and the phage morphology was observed by TEM (JEM-1200EX II, JEOL, Japan) at an acceleration voltage of 80 kV.

### 2.4 Optimal multiplicity of infection (MOI) determination

The host *R. anatipestifer* R-21 was cultured until it reached the logarithmic growth phase and was then adjusted to a concentration of 10^8^ CFU/mL. Phage GDF21 was incubated with *R. anatipestifer* R-21 at various proportions (0.0001, 0.001, 0.01, 0.1, 1, and 10). After cultivation at 37 °C for 12 h, the phage titer for each experimental condition was assessed using the double-layer agar plate method. The experiment was conducted in triplicate. The proportion with the highest phage titer was considered as the optimal MOI.

### 2.5 One-step growth curve

To understand the reproductive characteristics of phage GDF21, the eclipse period and burst size were assessed via a one-step growth curve assay. Briefly, phage GDF21 was mixed with *R. anatipestifer* R-21 at an MOI of 0.1 and incubated at 37 °C for 5 min, followed by centrifugation to eliminate unabsorbed phages. The resulting precipitate was resuspended in 20 mL of TSB after washing, and the phage titer was measured at 10-min intervals utilizing the double-layer agar plate method. The time interval between the initial adsorption of the phage and the onset of bacterial lysis was defined as the eclipse period (Chen D. et al., 2024). The burst size of phage was calculated by dividing the final count of newly produced phages by the initial phage titers (Chen D. et al., 2024).

### 2.6 Thermal and pH stability

To evaluate thermal stability, phage suspensions were prepared at a volume of 500 μL per tube and subjected them to incubation in a water bath set to temperatures of 40 °C, 50 °C, 60 °C, 70 °C, and 80 °C (Wan et al., 2025). Following these temperature exposures, the phage titer was measured at intervals of 20, 40, and 60 min using the double-layer agar plate method. This experiment was conducted three times.

In examining the stability of the phages across different pH levels, the phage suspensions were incubated at a range of pH levels from 1 to 13 at 37 °C for 1 h, after which the phage titers for each sample were measured employing the double-layer agar plate method. Each treatment was performed in triplicates (Wan et al., 2025).

### 2.7 Genome sequencing and bioinformatics analysis

The genomic DNA of phage GDF21 was extracted with the E.Z.N.A.^®^ Viral DNA kit (OMEGA, United States), followed by sequencing conducted by Shanghai Biozeron Biotechnology Co., Ltd., (Shanghai, China) utilizing the Illumina NovaSeq6000 sequencing platform to generate 150 bp paired-end reads (PE150). Raw reads underwent quality control with FastQC v0.12^1^ and contigs were assembled using Unicycler v0.5.0. Open reading frames (ORFs) were predicted using the GeneMarkS v4.28^2^, functional annotation was performed using the BLASTP^3^, and potential tRNA genes were predicted using tRNAscan-SE v2.0 (Chan et al., 2021). The presence of antibiotic resistance genes from the genomic data was analyzed using both comprehensive antibiotic resistance database (CARD)^4^ and ResFinder^5^ (Bortolaia et al., 2020; Alcock et al., 2023). The virulence genes were predicted in the virulence factor database (VFDB)^6^ (Chen et al., 2005; Zhu et al., 2025). The phage genome was visualized by Proksee^7^ (Grant et al., 2023).

The complete genomes of all 16 Riemerella phages were retrieved from the GenBank database and subsequently analyzed using VICTOR for phylogeny and classification (Meier-Kolthoff and Goker, 2017). Pairwise intergenomic similarities among the viral genomes were computed using the Virus Intergenomic Distance Calculator (VIRIDIC)^8^. Phylogenetic trees of phage GDF21 based on the terminase large subunit, major capsid protein, and tail protein were generated using the neighbor-joining method with the default parameters available in MEGA 11. Comparative genomic analysis was performed using the Mauve 20150226 (Darling et al., 2004). The whole genome sequences of phage GDF21 and phage vB_RanS_CRP19, which exhibited the highest homology, were compared using Easyfig.

### 2.8 Expression and purification of LysGDF21

The structure of LysGDF21 (encoded by orf 65) was analyzed by SWISS-MODEL^9^ and Phyre 2^10^. The endolysin gene LysGDF21 was amplified from the genomic DNA of phage GDF21 through PCR (Primer sequences: LysGDF21-F, 5′-CGACATATGTACCTGAAAACCTTTC-3′; LysGDF21-R, 5′-AGACTCGAGTTAGCGCATATCAT-3′), and then cloned into the pET-28a vector to yield recombined plasmids pET-28a-LysGDF21. This recombinant plasmid was subsequently transformed into E. coli BL21 (DE3) for the purpose of protein expression. *E. coli* BL21 (DE3) carrying pET-28a-LysGDF21 was grown in 200 mL of LB medium supplemented with kanamycin (30 μg/mL, Solarbio, China) at 37 °C until the optical density (OD) at 600 nm (OD_600_) reached between 0.6 and 0.8. Following this, 0.1 mM IPTG was added to induce expression at 28 °C for a duration of 16 h. The cultured cells were harvested and resuspended in PBS, after which sonication was performed on ice. After centrifugation, the soluble fraction was purified using a His-tagged protein purification kit (CWBIO, China) according to the instructions provided by the manufacturer. Following purification from a 1 L culture of *E. coli* BL21(DE3)/pET-28a-LysGDF21, we obtained 4 mL of the protein at a concentration of 2.5 mg/mL. The purified proteins were analyzed by SDS-PAGE using a 12% polyacrylamide gel containing 0.1% SDS. Following electrophoresis, the gel was stained with Coomassie Brilliant Blue R-250 for 1 h and subsequently destained with a solution of 10% acetic acid and 40% methanol until the background was clear and protein bands were visible. Protein concentration was determined using a BCA Protein Assay Kit (Solarbio, China).

### 2.9 Biofilm assay

The biofilm formation of *R. anatipestifer* was evaluated using the 96-well microtiter plate assay with minor modifications (Hu et al., 2010; Wang et al., 2023). Briefly, *R. anatipestifer* strains were diluted in sterile TSB medium at a final concentration of 10^8^ CFU/mL. A volume of 200 μL from each diluted culture was pipetted into the wells of 96- well microtiter plate and incubated at 37 °C in 5% CO_2_ for 48 h. A separate set of wells was filled with 200 μL of fresh TSB medium, which served as a negative control. Wells were then washed three times with PBS and fixed using methanol for 15 min. After the plates were allowed to air dry, they were stained with a 1% (w/v) crystal violet solution at room temperature for 30 min. Then, removed the crystal violet and washed the plate three times with sterile PBS. To dissolve the dye attached to the biofilm, a solution of 33% (v/v) glacial acetic acid was introduced to each well. The OD_595_ of each well was recorded with a microplate reader (Multiskan FC, Thermofisher, USA). Each experimental setup was conducted in triplicate, and the results are expressed as mean ± SD.

### 2.10 Inhibitory effect of LysGDF21 on biofilm formation

The activity of lysGDF21 to inhibit biofilm formation was investigated based on previous publication with minor modifications (Shahed-Al-Mahmud et al., 2021). Briefly, the *R. anatipestifer* strains were diluted in sterile TSB at a final concentration of 10^8^ CFU/mL. A total of 200 μL of each diluted culture mixed with 500 ng LysGDF21 was added to each well of 96- well microtiter plate and incubated at 37 °C in 5% CO_2_ for 48 h. The PBS was set as the control and the experiments were in triplicate. Biofilm formation was assessed using crystal violet staining as describe above.

### 2.11 Disruption of *R. anatipestifer* biofilms by LysGDF21

For elucidating the activity to disrupt biofilm, the biofilms formed by *R. anatipestifer* were washed with PBS and treated with 200 μL of LysGDF21 (500 ng) for 12 h, while an equal volume of PBS was used as the control. Each group contained triplicate samples. After incubation for 12 h, biofilms were assessed using crystal violet staining as described above.

### 2.12 Antibacterial activity assay of LysGDF21

The *R. anatipestifer* strains were cultured to the logarithmic phase and pretreated with 50 mM EDTA for 5 min. Then, bacterial cells were washed twice and resuspended in PBS to reach an OD_600_ ∼ 0.5. Each aliquot (180 μL) of bacterial suspension was added into sterile 96-well microtiter plates containing 20 μL of various concentrations of endolysin LysGDF21 (50, 100, and 500 ng). Followed by incubation at 37 °C in 5% CO_2_, the absorbance at OD_600_ was measured using a microplate reader (Multiskan FC, Thermofisher, USA) at different time points (0, 1, 2, 5, 12 h). For negative controls, PBS was added instead of endolysin. All the experiments were repeated three times.

### 2.13 Statistical analyses

All experiments were performed with at least three biological replicates under identical conditions. The data were analyzed using GraphPad Prism Version 8.3.0 software and presented as the mean ± standard deviation (SD). Statistic significances in each assay were assessed by Student’s *t*-test, where the *P*-values < 0.05 were considered statistically significant (*P < 0.05; **P < 0.01; and ***P < 0.001).

## 3 Results

### 3.1 Isolation and morphological characteristics of phage vB_RanS_GDF21

**FIGURE 1:**
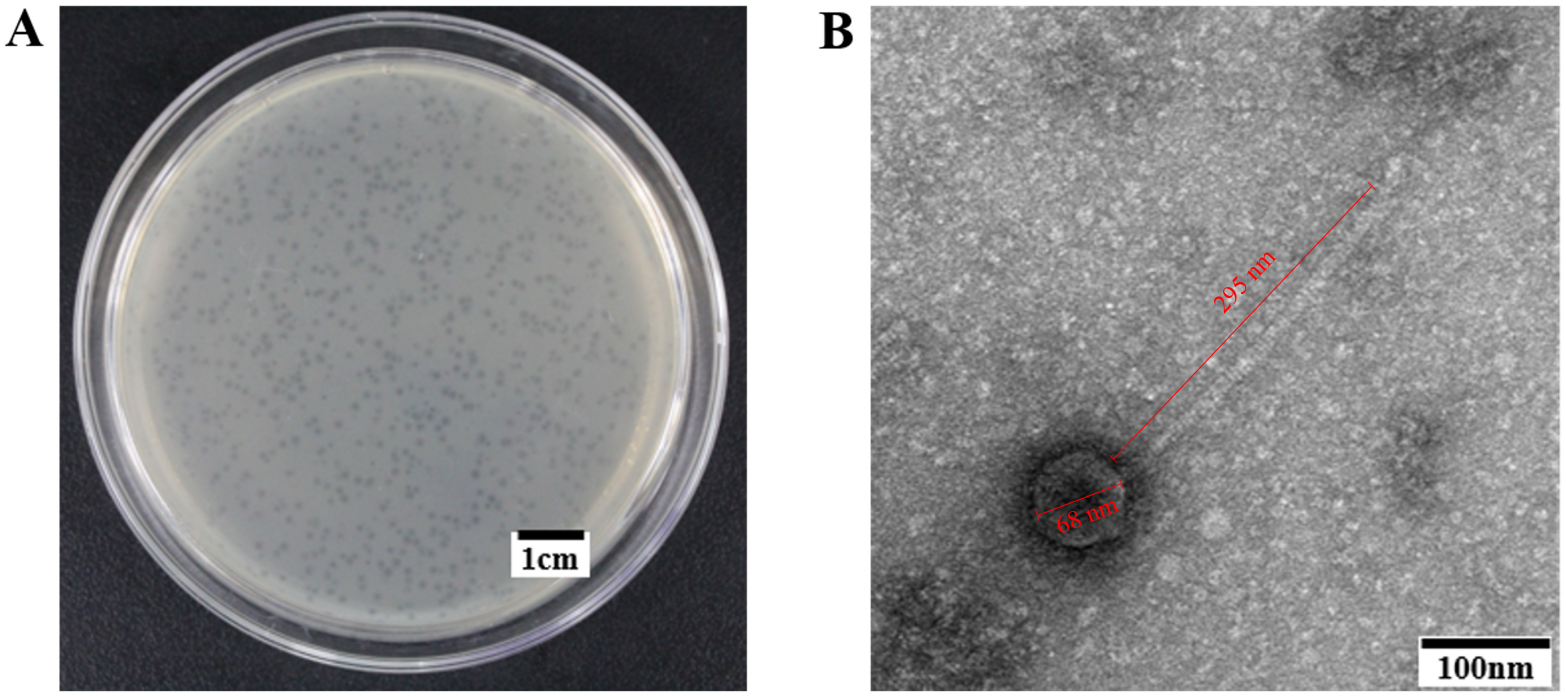
Morphology of phage GDF21. **(A)** Phage plaques formed of phage GDF21 on the plate with *R. anatipestifer* R-21. The scale bar is 1 cm. **(B)** Phage GDF21 was stained negatively and observed by transmission electron microscope (TEM). GDF21 had an icosahedral symmetric head approximately 68 nm in diameter, and a long, non-contractile tail of approximately 295 nm length. Scale bar, 100 nm.

In this research, a novel *R. anatipestifer* phage, named vB_RanS_GDF21 (GDF21), was isolated from 36 duck feces samples as described in see section “2.2 Isolation and purification of phage.” The purification of the phage was conducted using the double-layer agar plate method, revealing the formation of transparent, round plaques with diameters nearing 0.8 mm on the plate (Figure 1A). TEM revealed that phage GDF21 had an icosahedral symmetric head approximately 68 nm in diameter, and a long, non-contractile tail of approximately 295 nm length (Figure 1B).

### 3.2 Biological characterization of phage vB_RanS_GDF21

**FIGURE 2:**
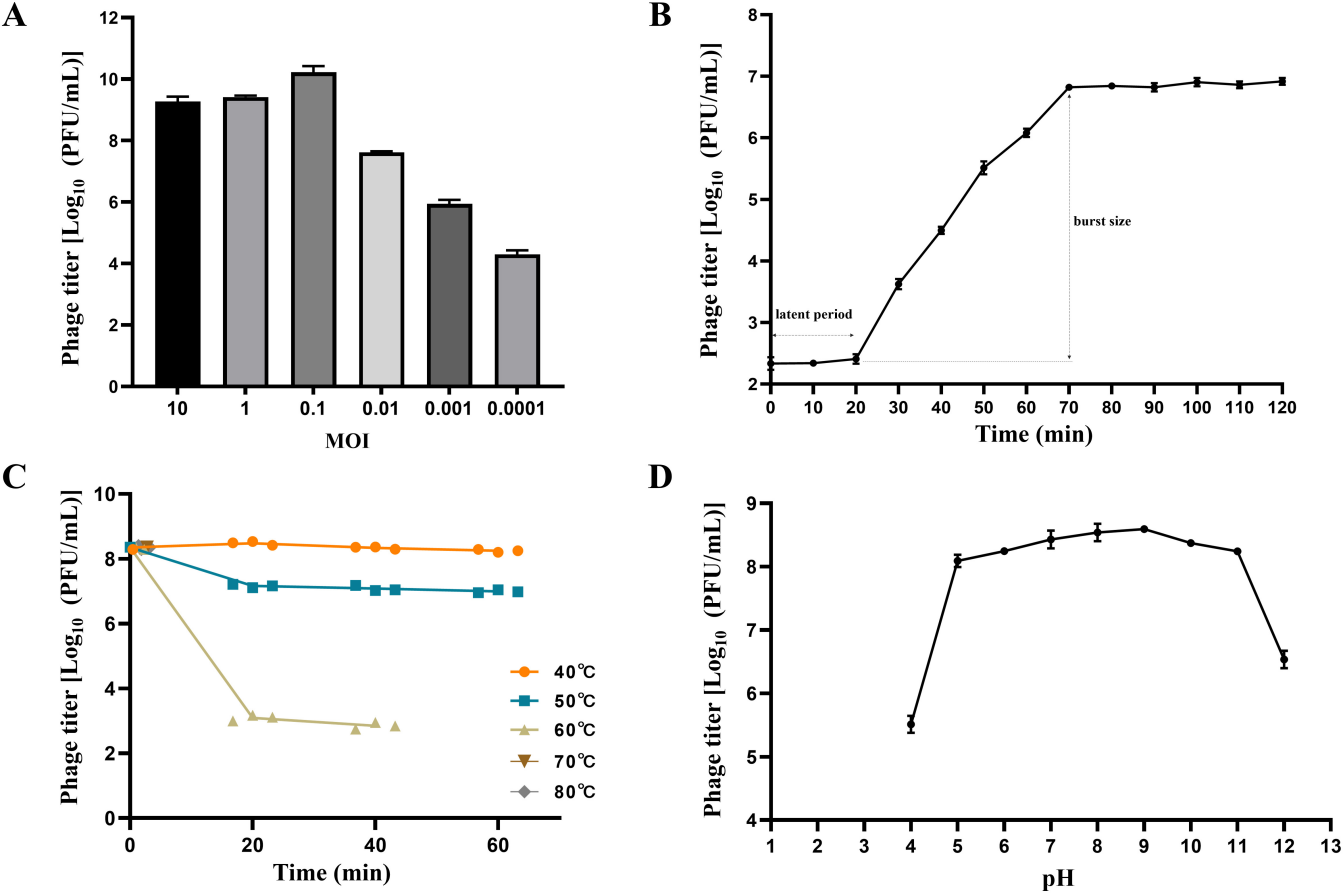
Biological characterization of phage GDF21. **(A)** Optimal MOI of phage GDF21. **(B)** One-step growth curve of phage GDF21 on *R. anatipestifer* R-21. **(C)** Thermal stability. **(D)** pH stability of phage GDF21. Data represent mean ± SD (*n* = 3 biological replicates).

Phage GDF21 exhibited the highest titer of 1.8 × 10^10^ PFU/mL on *R. anatipestifer* R-21 at the MOI of 0.1, suggesting that an MOI of 0.1 is optimal for the infection of GDF21 (Figure 2A). A one-step growth curve analysis was conducted to assess the lytic cycle of phage GDF21, revealing that the eclipse period lasted approximately 20 min, followed by a rapid release of viral particles until 70 min, and then remained stable (Figure 2B). The burst size of GDF21 was found to be around 74 PFU/infected cell. The stability of phage GDF21 was tested under different temperature and pH conditions. The thermal stability assessments indicated that GDF21 remained generally stable at temperatures below 50 °C, albeit there was a slight reduction in titer at 50 °C. Moreover, the phage’s activity was completely compromised at 60 °C after a duration of 40 min, and no phage was detected at 70 °C and 80 °C (Figure 2C). In the pH stability test, phage GDF21 was observed to be stable in the pH ranging from 5 to 11, yet prominently inactivated under extreme acidic and alkaline conditions, such as below pH 3 or above 13 (Figure 2D).

### 3.3 Genome analysis of phage vB_RanS_GDF21

**FIGURE 3:**
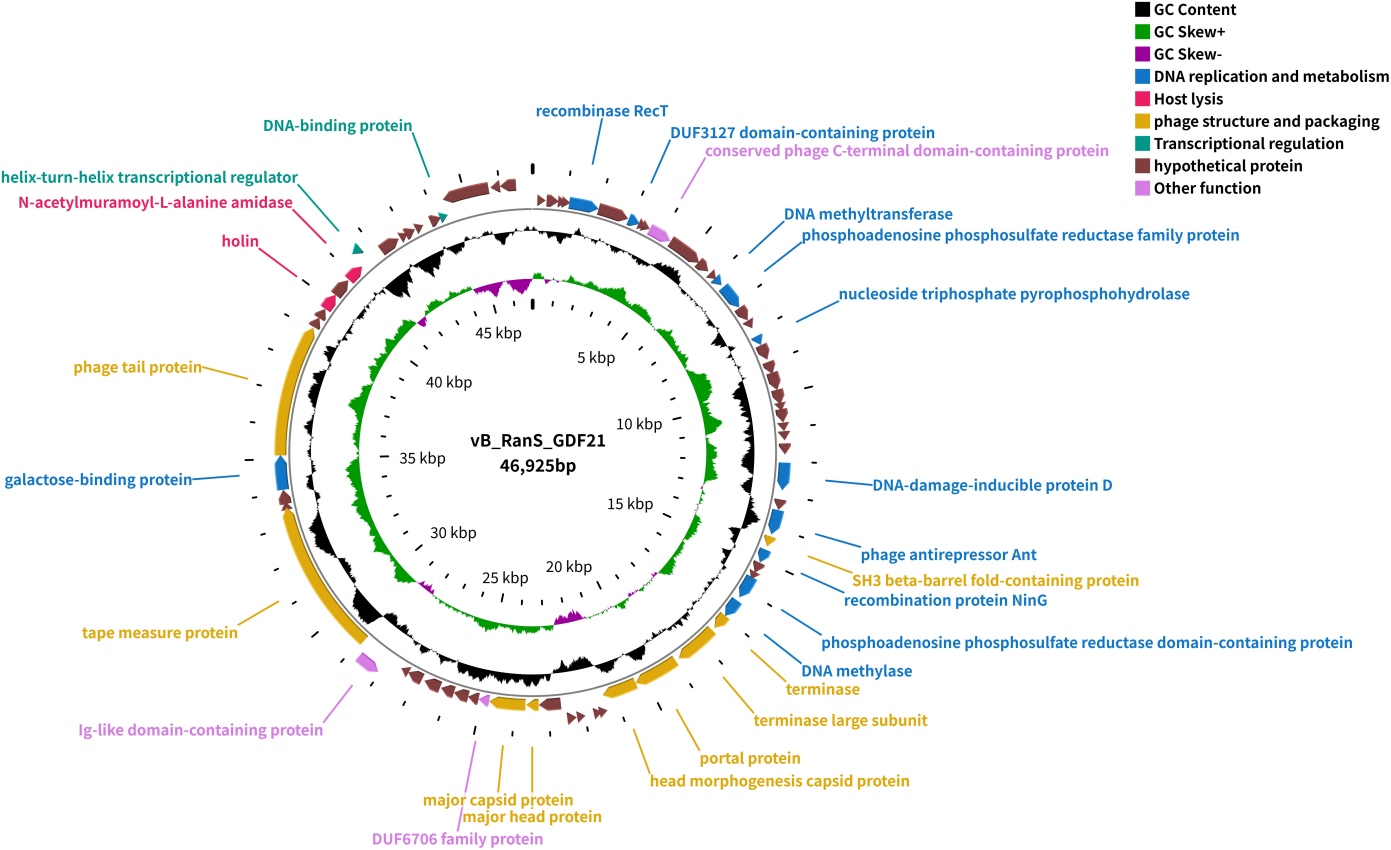
Circular genome map of phage GDF21. The outer circle represents 75 ORFs, with clockwise and counterclockwise arrows indicating forward and reverse transcriptional directions. The predicted functional proteins are color-coded by functional category. The inner circle shows GC skew [(G−C)/(G+C)] (green/purple), while the middle circle plots GC content (black).

Whole genome sequencing analysis revealed that the genome of phage GDF21 consists of a 46,925 bp double-stranded DNA molecule with a G/C content of 35.25% (Figure 3). The GeneMarkS analysis demonstrated that a total of 75 ORFs were predicted in the genome of phage GDF21, 27 of which were annotated as functional genes by the BLASTP analysis. The predicted functional proteins primarily involved in DNA replication and metabolism, host lysis, phage structure and packaging, and transcriptional regulation. The lysis system of phage GDF21 was identified as a canonical two-component system, where ORF63 encodes a holin and the adjacent ORF65 encodes the endolysin (LysGDF21) (Figure 3). In addition, the genome of phage GDF21 revealed the absence of tRNA, virulence, and antibiotic resistance genes. The annotations in detail were given in Supplementary Table 1, and the whole genome was visualized in Figure 3. The whole genome sequencing data of GDF21 can be accessed from GenBank with the accession number PP067979.

### 3.4 Phylogenetic and comparative genomic analysis

**FIGURE 4:**
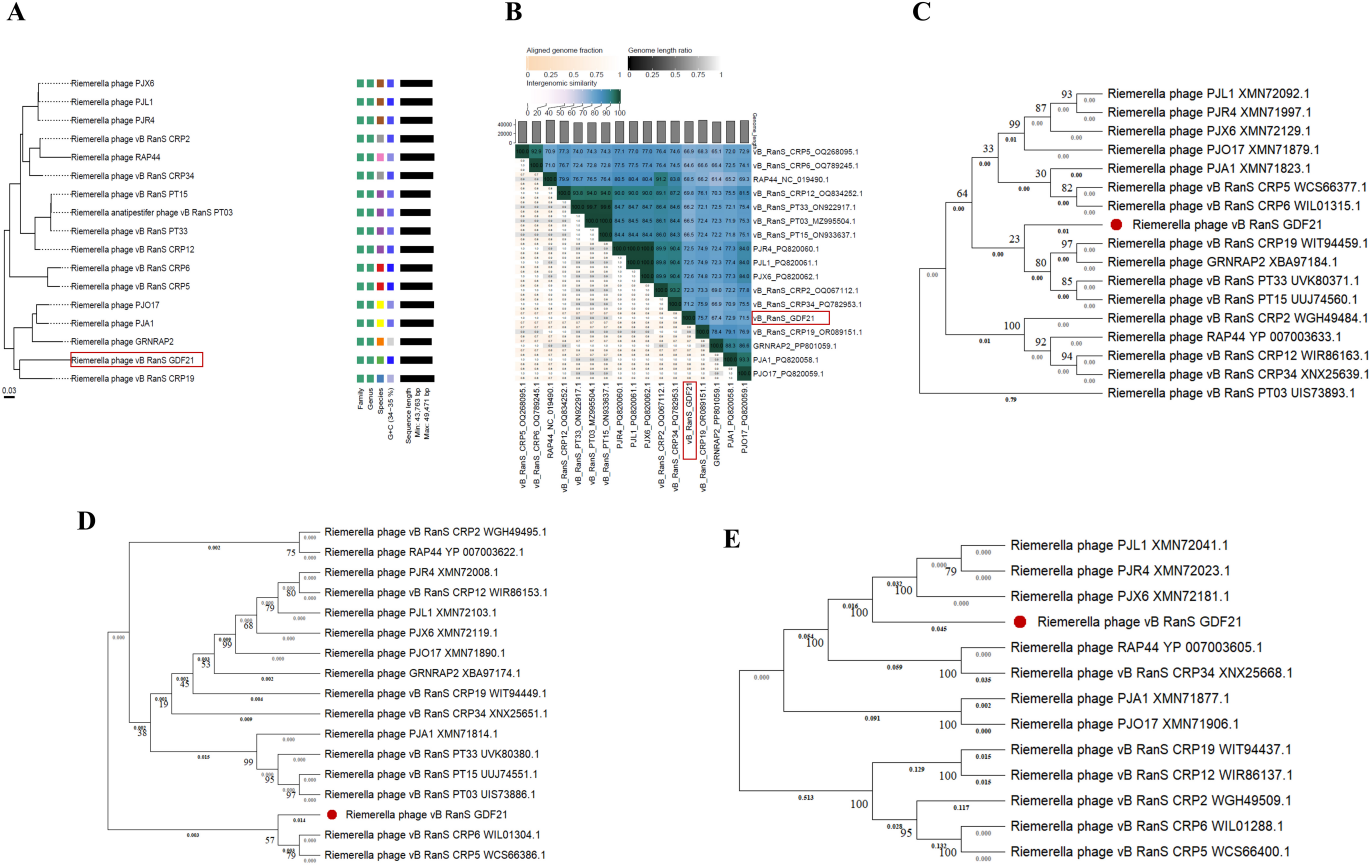
Phylogenetic analysis of phage GDF21. **(A)** The phylogeny and classification were analyzed using VICTOR. The left half displays the phylogenetic tree based on whole genome sequences, while the right half shows the classification according to the ICTV. **(B)** Heatmap of intergenomic similarity between phage GDF21 and its related phages was generated using VIRIDIC. The position of GDF21 is highlighted with a red box. Phylogenetic trees of the terminase large subunit **(C)**, major capsid protein **(D)**, and tail protein **(E)** were constructed using MEGA 11, with the red filled circle in each tree highlighting the position of phage GDF21 identified in this work.

The VICTOR analysis indicated that all examined *Riemerella* phages cluster within the same genus, which currently lacks an official taxonomic classification (Figure 4A). The phylogenetic tree showed that phage GDF21 is most closely related to phage vB_RanS_CRP19 (59.87% similarity) (Figure 4A). Given that the phage vB_RanS_CRP19 is classified within the class *Caudoviricetes*, it is highly probable that phage GDF21 also belongs to the *Caudoviricetes* class. Intergenomic similarity analysis using VIRIDIC revealed that phage GDF21 shares the highest similarity with phage vB_RanS_CRP19 (OR089151.1) at 75.7%, followed by phage PJA1 (PQ820058.1) at 72.9% (Figure 4B). Furthermore, phylogenetic trees were constructed based on the terminase large subunit, major capsid protein, and tail protein. In the tree of the terminase large subunit, GDF21 formed a distinct cluster with vB_RanS_CRP19 (WIT94459.1), GRNRAP2 (XBA97184.1), vB_RanS_PT33 (UVK80371.1), and vB_RanS_PT15 (UUJ74560.1), indicating a close phylogenetic relationship (Figure 4C). The major capsid protein tree showed that GDF21 is most closely related to CRP6 (WIL01304.1) and CRP5 (WCS66386.1) (Figure 4D), while the tail protein tree revealed that GDF21 clusters most closely with PJL1 (XMN72041.1), PJR4 (XMN72023.1), and PJX6 (XMN72181.1) (Figure 4E).

**FIGURE 5:**
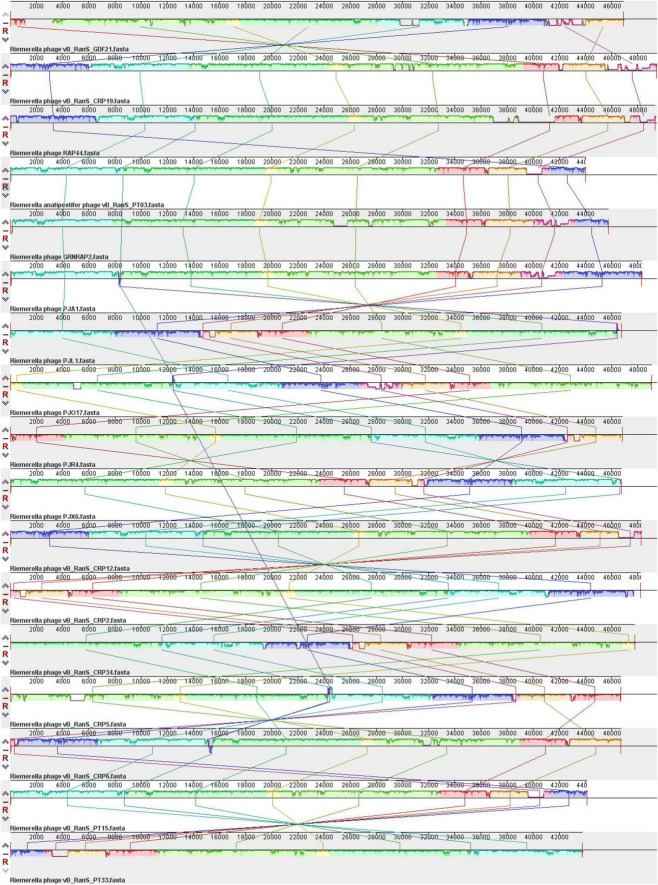
Multiple genome alignment of phage GDF21 using Mauve software. Each genome is laid out in a horizontal track. The colored blocks encompass similar regions between the phage genomes, and the height of the plates inside the blocks reflects the intensity of nucleotide similarity. Areas that exhibit a deficiency in homology are located outside these blocks or depicted in white inside the blocks.

**FIGURE 6:**
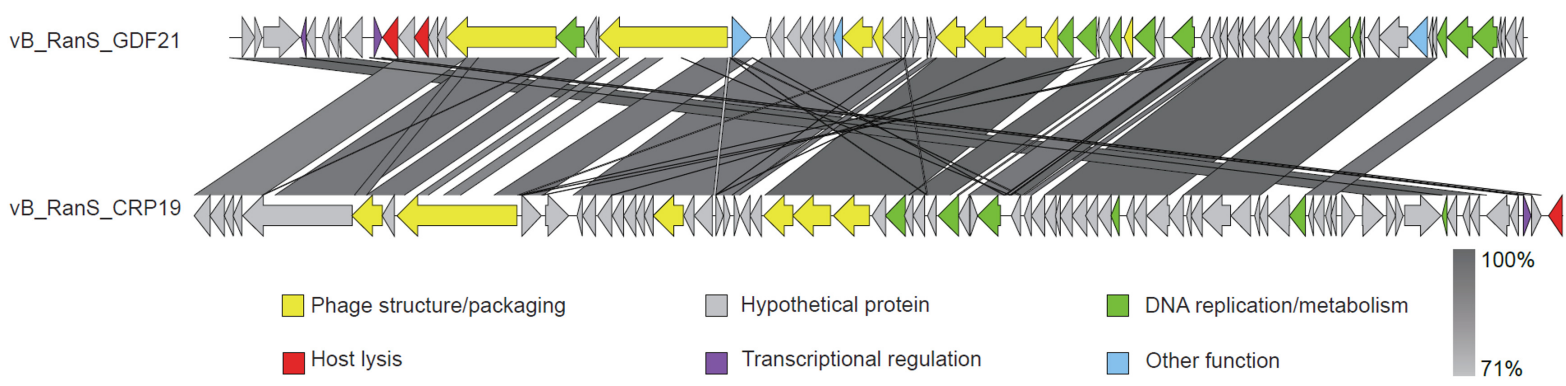
Comparative analysis of phage GDF21 and vB_RanS_CRP19 using Easyfig. The colored arrows indicate the predicted ORFs and transcriptional directions. The homologous regions between the two phages are highlighted with gray shading.

Comparative analyses of complete genome sequences between phage GDF21 and other phages were performed using Mauve. The results demonstrated multiple locally collinear blocks (LCBs) between GDF21 and other *Riemerella* phages, which were assigned the same color (Figure 5). This indicates that these phages share a similar genome organization, although some rearrangements and inversions were identified. To further investigate, the relatedness of GDF21 to the phage with the highest homology, vB_RanS_CRP19, was assessed using Easyfig software (Figure 6). The sections that displayed the most similarities included phage structure/packaging proteins and DNA replication-associated proteins.

### 3.5 Identification and expression of LysGDF21

**FIGURE 7:**
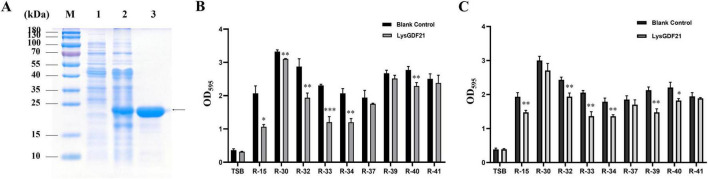
Effects of LysGDF21 on biofilm inhibition and disruption in *R. anatipestifer*. **(A)** Expression and purification of LysGDF21 analyzed by SDS-PAGE. Lane M, molecular weight markers; Lane 1, lysate of IPTG induced BL21(DE3) cells harboring backbone plasmid; Lane 2, lysate of IPTG induced BL21(DE3) cells harboring pET-28a-*LysGDF21*; Lane 3, purified LysGDF21. **(B)** Inhibition of biofilm formation. **(C)** Disruption of preformed biofilms by LysGDF21 (2.5 μg/mL, 12 h) in nine *R. anatipestifer* isolates. Data are presented as mean ± SD (*n* = 3). *, *p* < 0.05; **, *p* < 0.01; ***, *p* < 0.001.

By mining the genome data of GDF21, the *orf 65* was predicted to produce a putative endolysin with the *N*-acetylmuramoyl-L-alanine amidase activity indicated by SWISS-MODEL and Phyre 2. The predicted *endolysin* gene (named *LysGDF21*) was amplified by PCR then cloned into the plasmid pET-28a for expression in *E. coli* BL21. The LysGDF21 was purified by affinity chromatography and detected by SDS-PAGE. The results showed that the recombined proteins were expressed in soluble forms with mass of approximately 22.75 kD (Figure 7A).

### 3.6 Anti-biofilm and antibacterial effects of LysGDF21

Endolysins produced by phages often exhibit a broader spectrum comparing to their host phages (Schuch et al., 2002; Li et al., 2021; Wang F. et al., 2022). However, their efficacy against Gram-negative bacteria is often dependent on combination with outer membrane permeabilizers. Given their potent antimicrobial properties, we further investigated the antibacterial and anti-biofilm activity of the endolysin LysGDF21.

Initially, the biofilm formation capability of 51 clinically isolated *R. anatipestifer* strains was assessed using crystal violet staining. Based on the results shown in Supplementary Figure 1, a total of nine strong biofilm-forming strains (R-15, R-30, R-32, R-33, R-34, R-37, R-39, R-40, and R-41) with OD_5_95 values ≥ 2.0 were selected for subsequent experiments.

To evaluate the inhibitory effect of LysGDF21 on biofilm formation, the nine selected *R. anatipestifer* strains were pretreated with LysGDF21 (2.5 μg/mL) prior to incubation, thereafter the biofilms were quantified using crystal violet staining at 48 h post treatment. Our results demonstrated that LysGDF21 significantly inhibited biofilm formation of *R. anatipestifer* strains R-15, R-30, R-32, R-33, R-34, and R-40 (*p* < 0.05), with the most inhibitory effects observed in R-15 (48.6% reduction) and R-33 (47.8% reduction). However, this inhibition was less pronounced against strains R-37, R-39, and R-41 (Figure 7B). To assess the biofilm disruption capability of LysGDF21, *R. anatipestifer* biofilms were treated with LysGDF21 (2.5 μg/mL) for up to 12 h. The results indicated that LysGDF21 significantly eliminated the biofilm formed by *R. anatipestifer* strains R-15, R-32, R-33, R-34, R-39, and R-40 (*p* < 0.05). The greatest biofilm disruption was observed in strains R-33 (33.5% decrease) and R-39 (30.6% decrease). In contrast, LysGDF21 treatment exhibited no activity against biofilm for strains R-30, R-37, and R-41 (Figure 7C). Our findings revealed that LysGDF21 effectively inhibited biofilm formation and disrupted preformed biofilms in *R. anatipestifer.*

**FIGURE 8:**
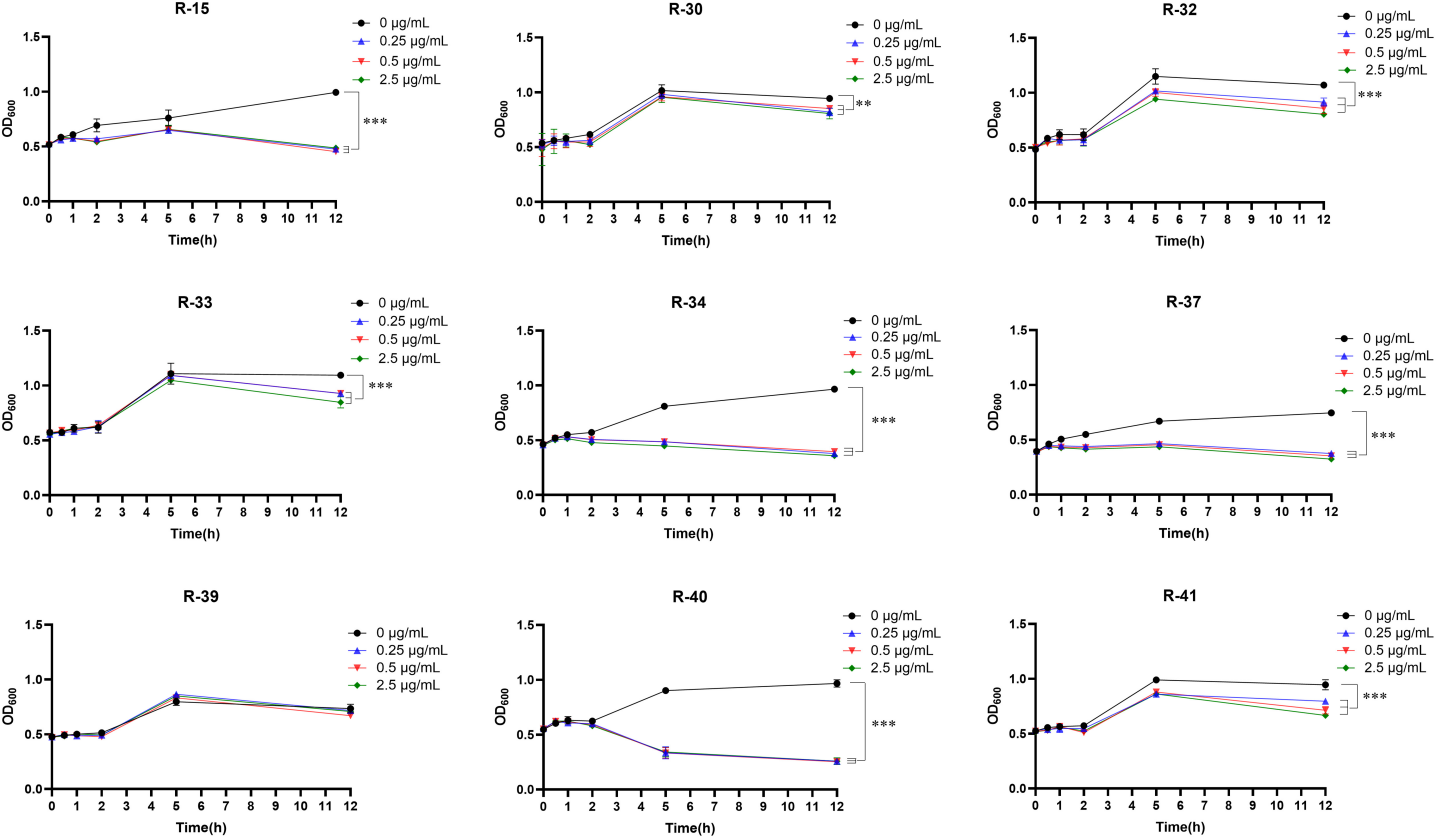
Lytic activity assay of LysGDF21 against *R. anatipestifer*. Nine *R. anatipestifer* strains (R-15, R-30, R-32, R-33, R-34, R-37, R-39, R-40, R-41) pretreated with 50 mM EDTA were treated with LysGDF21 at final concentrations of 0.25, 0.5, and 2.5 μg/mL. PBS-treated samples served as untreated controls. Antibacterial activity was quantified by monitoring OD_600_ at 0, 1, 2, 5, and 12 h post-treatment. Data are expressed as mean ± SD (*n* = 5). **, *p* < 0.01; ***, *p* < 0.001.

Subsequently, we elucidated the antibacterial activity of LysGDF21 against the aforementioned nine *R. anatipestifer* strains, however, no lytic activity was observed throughout all experiments (data not shown). We assumed that it was because LysGDF21 was not able to penetrate the outer membrane to access the peptidoglycan layer. The studies in precedence have reported that EDTA can enhance the lytic activity of endolysins against Gram-negative bacteria by permeabilizing the outer membrane (Wang X. et al., 2022). In this regard, the antibacterial activity of LysGDF21 was evaluated against *R. anatipestifer* in presence EDTA for penetration. As shown in Figure 8, LysGDF21 treatment (50, 100, and 500 ng) significantly inhibited the growth of all tested *R. anatipestifer* strains (*p* < 0.01), with the exception of strain R-39. Particularly, the viability (shown as OD_600_) of R-15, R-34, and R-40 sharply decreased after LysGDF21 treatment over 12 h, indicating the lytic actions were exerted by the LysGDF21 (Figure 8). Additionally, strains R-32 and R-41 displayed dose-dependent susceptibility to LysGDF21. These results showed that LysGDF21 was actively lytic against *R. anatipestifer* in combination with EDTA.

Our findings demonstrate that LysGDF21 explicated significant anti-biofilm and antimicrobial activity against several *R. anatipestifer* strains, highlighting its potential as a promising therapeutic alternative for controlling *R. anatipestifer* infections in poultry industry.

## 4 Discussion

*R. anatipestifer* is a Gram-negative bacterium that belongs to the *Riemerella* genus within *Flavobacteriaceae* family (Wu et al., 2024). It is an important pathogen to poultry and wild birds. Infected ducks develop septicemia, meningitis, and infectious serositis (Zou et al., 2023). Several studies have documented *R. anatipestifer* infections in chickens, with clinical manifestations primarily characterized by salpingitis and arthritis (Omaleki et al., 2021; Chen Y. et al., 2024). These diseases result in high mortality rates and substantial economic losses in the poultry industry worldwide (Hao et al., 2025). *R. anatipestifer* exhibits complex serological diversity, with at least 21 identified serotypes demonstrating limited cross-protection by vaccines (Huang et al., 2023). Furthermore, the increasing emergence of antibiotic resistance in *R. anatipestifer* severely compromises the efficacy of antibiotic treatments (Zhu et al., 2023). Although phages and their associated proteins (such as endolysin and depolymerase) represent an effective alternative strategy against bacterial infections, particularly for multidrug-resistant strains, combating *R. anatipestifer* using phage remains poorly explored. To date, only one phage (RAP44) has been previously characterized (Cheng et al., 2012; Wang Y. et al., 2022). In this study, we isolated and characterized a new *R. anatipestifer* phage, GDF21, with comprehensive analysis on its biological characteristics, genomic features, and the antimicrobial activity of its encoded endolysin.

The *R. anatipestifer* phage GDF21 exhibits typical siphophage morphology, with similarity to the previously reported *R. anatipestifer* phage RAP44 (Cheng et al., 2012). The determination of the optimal MOI for phages is critical to its production and utilization in scale (Abedon, 2016). In the present study, phage GDF21 exhibited optimal replication at an MOI of 0.1, yielding a high titer of 1.8 × 10^10^ PFU/mL. These findings suggest that GDF21 is supposed to elicit maximal antibacterial efficacy under clinically relevant conditions. It has been established that high burst size and short eclipse period are positively correlated with bacterial eradication efficacy (Abedon et al., 2001). Phage GDF21 presented an eclipse period of 20 min and an average burst size of 74 PFU/cell, indicating favorable host infectivity and effective lytic activities. However, to our knowledge, limited data are available regarding the one-step growth curve characteristics of *R. anatipestifer* phages in current literatures. The lack of comparative data limits a thorough discussion of replication kinetics among different *R. anatipestifer* phages. It has been well acknowledged that the tolerances to temperature and pH are essential for phage activity (Jonczyk-Matysiak et al., 2019). GDF21 exhibited broad pH tolerance and good thermal stability. There is no significant reduction observed under either acidic or alkaline conditions (pH 5-11). Furthermore, phage GDF21 also survived in the temperature below 50 °C for at least 60 min. These properties highlight its potential for biocontrol applications against *R. anatipestifer* infections.

The genomic and phylogenetic analysis of phage GDF21 provide critical insights into its evolutionary relationships and genomic diversity within the related phage group (Shang et al., 2021; Cao et al., 2024). A total of 75 ORFs were identified in the phage GDF21 genome, where only 27 ORFs have been annotated with specific functions. The annotation suggests that the GDF21 genome encodes the genes associated with DNA replication and metabolism, lysis-related protein, phage structure and packaging, and several regulatory proteins responsible for transcriptional regulation. The absence of tRNA, virulence, or antibiotic resistance genes supports its potential utility for practice (Loc-Carrillo and Abedon, 2011). Phylogenetic and classification analyses showed that phage GDF21 is closely related to *Riemerella* phage vB_RanS_CRP19 and is classified within the class *Caudoviricetes*. Comparative genomic analysis revealed conserved syntenic blocks and structural variations between GDF21 and other known *Riemerella* phages, indicating shared evolutionary origin and genomic plasticity. Notably, the presence of divergent loci suggests acquisition or loss of genetic elements in these *Riemerella* phages. These findings align with prior studies on phage evolution (Tie et al., 2018; Cong et al., 2021). Further studies are warranted to elucidate the biological implications of these genetic variations for phage adaptability and host specificity.

Based on our bioinformatics predictions, the *orf 65* of GDF21 (LysGDF21) most likely encodes a endolysin. The LysGDF21 was predicted to be a modular endolysin consist of one *N*-acetylmuramoyl-L-alanine amidase catalytic domain which is able to degrade the peptidoglycan by cleaving the amide bond between *N*-acetylmuramoyl and L-amino acids (Abdelrahman et al., 2021).

Bacterial biofilms are composed of polymeric substances secreted by bacteria, which confer protection to embedded cells against antibacterial disinfectants (Davies, 2003). Biofilm formation has been well-documented in *R. anatipestifer* and various other pathogens (O’Toole et al., 2000; Hu et al., 2010). Substantial evidence indicates that biofilms are considered as the primary causes of resistance mechanisms, leading to persistent or recurrent infections and consequently increasing the difficulty of treatments (O’Toole et al., 2000; Lewis, 2005; Koo et al., 2017). Notably, phage endolysins demonstrate remarkable efficacy against bacterial biofilms (Koo et al., 2017; Sharma et al., 2018). Previous studies reported that endolysin LysCSA13 disrupts *S. aureus* biofilms (Cha et al., 2019). Recombinant *Staphylococcal* bacteriophage endolysin CHAP_k_ exhibited broad anti-biofilm properties against *S. aureus* and *Streptococcus agalactiae* (Shan et al., 2020). Abtn-4 had the ability to reduce biofilm formation of *A. baumannii* (Yuan et al., 2021). In this study, nine strong biofilm-forming *R. anatipestifer* strains were selected to evaluate the anti-biofilm activity of LysGDF21. Our findings demonstrated that LysGDF21 displays significant anti-biofilm activity against *R. anatipestifer*. The greatest biofilm formation inhibition was observed against strain R-15 (48.6% reduction relative to untreated controls), whereas the most pronounced biofilm disruption efficacy was found against strain R-33 (33.5% decrease compared to control biofilms). The varied efficacies against different strains are likely stems from differences in biofilm matrix composition between strains. For instance, the recalcitrance of R-37/R-41 biofilms to LysGDF21 suggests the presence of structural barriers. LysGDF21 could only disrupt preformed mature biofilms of R-39 (30.6% reduction) implies that mature biofilms in this strain might expose vulnerable targets (e.g., unmasked peptidoglycan layers) not accessible during initial biofilm formation. The formation of biofilms is often dynamic and mature biofilms are less susceptible to antimicrobial agents than early-stage biofilms due to differences in metabolic activity and stress responses against antimicrobials (Ito et al., 2009; Chu et al., 2022). In our research, LysGDF21 exhibited higher efficacy in inhibiting biofilm formation (48.6% in R-15; 32.5% in R-32) than in disrupting preformed biofilms (23.8% and 20.3%, respectively), a trend consistent across R-33, R-34, and R-40 strains. These results demonstrate LysGDF21’s potential as a preventive agent against multiple biofilm-associated pathogens.

Phage-derived endolysins generally demonstrate a broader lytic spectrum than their original phages (Oliveira et al., 2014; Abdelrahman et al., 2021); however, no direct bactericidal activity was observed for LysGDF21 in our study. Although LysGDF21 is unlikely to penetrate the outer membrane of *R. anatipestifer*, and despite bacterial biofilms presenting a structure more complex than the outer membrane or LPS alone, our results demonstrate its significant anti-biofilm efficacy. We therefore hypothesize that this activity may be attributed to strain-specific variations in biofilm matrix composition. Furthermore, the dynamic nature of biofilm formation may transiently expose vulnerable targets, such as underlying peptidoglycan, potentially facilitating endolysin action (Ito et al., 2009; Chu et al., 2022). To date, few studies have reported endolysins with natural lytic activity against Gram-negative bacteria. In general, the activity of endolysins against Gram-negative bacteria is limited, since the outer membrane acts as a physical barrier preventing access of endolysin to the peptidoglycan (Hermoso et al., 2007; Abdelrahman et al., 2021). Several strategies to enhance outer membrane permeability in Gram-negative bacteria have been reported, including the use of EDTA (Gerstmans et al., 2016; Lai et al., 2020). Cui et al. (2023) reported that the combination of endolysin LysASP and EDTA exerted antimicrobial activity against *P. aeruginosa*. Lim et al. (2012) observed that the addition of EDTA with SPN1S endolysin allowed strong antimicrobial activity. In this study, EDTA was employed to destabilize the outer membrane of bacterial cells. Combined with EDTA, the recombinant endolysin LysGDF21 lysed 8/9 tested *R. anatipestifer* strains, demonstrating a broader lytic spectrum than its parent phage GDF21. These findings indicated that LysGDF21 represents a promising preventive or therapeutic strategy for *R. anatipestifer* control in poultry production.

Although LysGDF21 demonstrates antimicrobial efficacy against *R. anatipestifer*, its activity is strictly dependent on the presence of EDTA for outer membrane penetration. However, the clinical application of outer membrane permeabilizers like EDTA is limited by their significant toxicity, rendering them unsuitable for use in food/feed additives or systemic infection treatments (Abdelrahman et al., 2021). Therefore, identifying novel endolysins with intrinsic outer membrane-penetrating capabilities, along with developing engineered endolysins through genetic modification, represents a promising strategy with substantial therapeutic potential.

## 5 Conclusion

In this study, a novel *R. anatipestifer* phage, named vB_RanS_GDF21, was isolated and characterized. It belongs to the class *Caudoviricetes*, exhibiting high productivity with a short eclipse period, robust pH and thermal stability, a narrow host range, and absence of virulence or antibiotic resistance genes. Genomic and comparative genomic analyses revealed the genome architecture and evolutionary relationships of phage vB_RanS_GDF21. Furthermore, we demonstrated that the phage-derived endolysin LysGDF21 exhibited potent anti-biofilm activity and broad antimicrobial efficacy against *R. anatipestifer*. Collectively, this study expanded the *R. anatipestifer* phage library and identified LysGDF21 as a promising candidate for developing novel therapeutic agents against *R. anatipestifer* infections.

## Data Availability

The datasets presented in this study can be found in online repositories. The names of the repository/repositories and accession number(s) can be found in this article/Supplementary material.

## Footnotes

1
https://www.bioinformatics.babraham.ac.uk/projects/fastqc/

2
http://topaz.gatech.edu/GeneMark/genemarks.cgi

3
https://blast.ncbi.nlm.nih.gov/Blast.cgi?PAGE=Proteins

4
https://card.mcmaster.ca/

5
https://cge.food.dtu.dk/services/ResFinder/

6
http://www.mgc.ac.cn/cgi-bin/VFs/v5/main.cgi

7
https://proksee.ca

8
https://rhea.icbm.uni-oldenburg.de/viridic/

9
https://swissmodel.expasy.org/

10
https://www.sbg.bio.ic.ac.uk/phyre2/html/page.cgi?id=index
