# Supplementary material for: Characterization of a new lytic bacteriophage vB_RanS_GDF21 and its endolysin LysGDF21 with antimicrobial activity against Riemerella anatipestifer
Source: Front Microbiol. 2026 Jan 13;16:1715949. doi: 10.3389/fmicb.2025.1715949 (PMC12845321; doi:10.3389/fmicb.2025.1715949)
Supplement: Supplementary file 1 [file Data_Sheet_1.doc]

**Supplementary Figures**

**
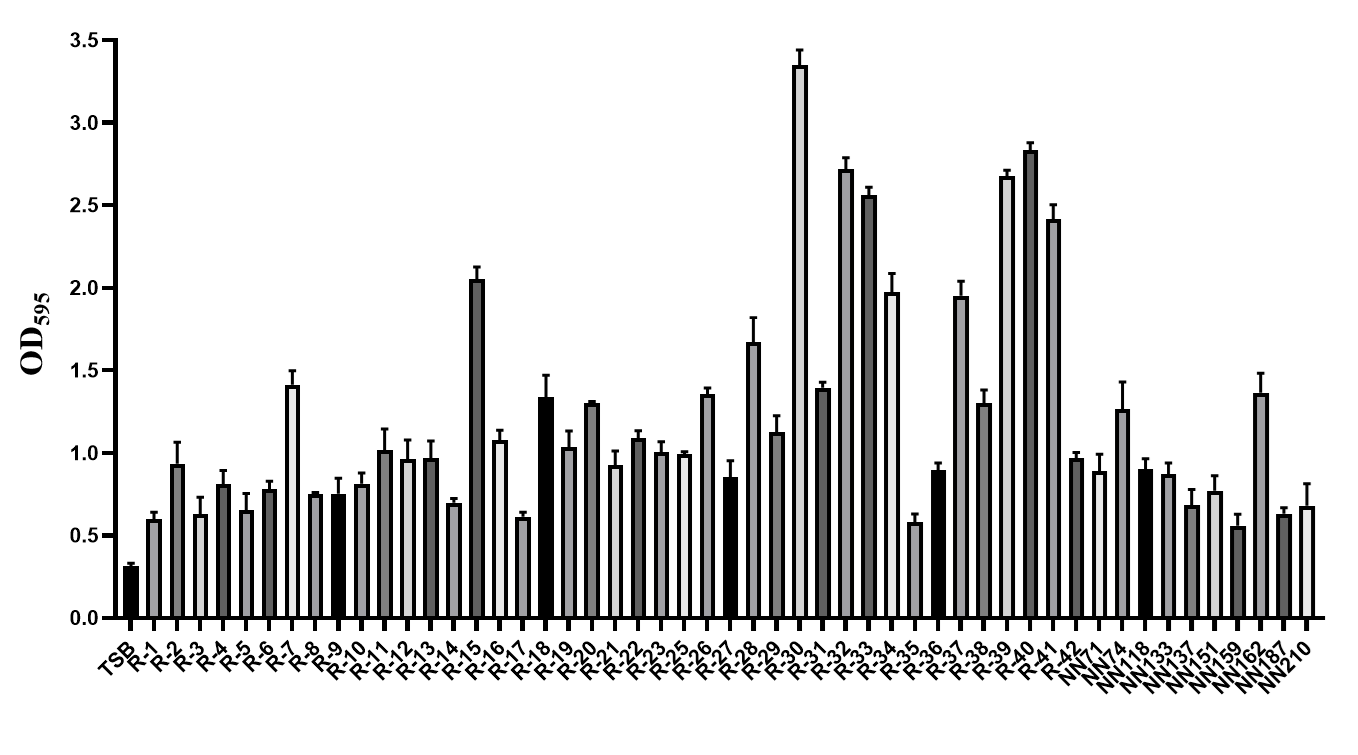
**

**Figure S1. Biofilm formation capacity of *R. anatipestifer* strains assessed by crystal violet staining assay.** The *R. anatipestifer* strains R-15, R-30, R-32, R-33, R-34, R-37, R-39, R-40, and R-41, which exhibited OD₅₉₅ values ≥ 2.0 were selected for subsequent experiments. Data represent mean values ± SD from three independent biological replicates.
