# Supplementary material for: Characterization of a new lytic bacteriophage vB_RanS_GDF21 and its endolysin LysGDF21 with antimicrobial activity against Riemerella anatipestifer
Source: Front Microbiol. 2026 Jan 13;16:1715949. doi: 10.3389/fmicb.2025.1715949 (PMC12845321; doi:10.3389/fmicb.2025.1715949)
Supplement: Supplementary file 2 [file Table_1.doc]

Supplementary Tables
Table S1. Genome annotation of phage GDF21.
ORF no.	Strand	Start	Stop	Length (AA)	Putative Function	Scientific Name	Query Cover	E value	Per. Ident	Accession	
1	+	148	381	77	hypothetical protein	Riemerella anatipestifer	87%	1e-37	95.52%	WP_064969687.1	
2	+	415	783	122	hypothetical protein	Riemerella anatipestifer	100%	6e-81	100.00%	WP_214194015.1	
3	+	786	971	61	hypothetical protein	Riemerella phage PJO17	100%	7e-33	80.33%	XMN71928.1	
4	+	946	1047	33	hypothetical protein	Riemerella phage PJA1	100%	8e-12	90.91%	XMN71856.1	
5	+	1088	1975	295	recombinase RecT	Riemerella anatipestifer	100%	0.0	99.32%	WP_317192685.1	
6	+	1975	2898	307	hypothetical protein	Riemerella anatipestifer	100%	0.0	99.35%	WP_262988307.1	
7	+	2918	3274	118	DUF3127 domain-containing protein	Riemerella anatipestifer	100%	2e-77	96.61%	WP_153937476.1	
8	+	3275	3454	59	hypothetical protein	Riemerella anatipestifer	100%	3e-32	94.92%	WP_153928880.1	
9	+	3408	3551	47	hypothetical protein	Riemerella anatipestifer	100%	8e-24	95.74%	WP_262987868.1	
10	+	3607	4311	234	conserved phage C-terminal domain-containing protein	Riemerella anatipestifer	100%	3e-167	99.15%	WP_264314764.1	
11	+	4324	5379	351	hypothetical protein	Riemerella anatipestifer	100%	0.0	99.43%	WP_013447258.1	
12	+	5354	5755	133	hypothetical protein	Riemerella anatipestifer	100%	1e-93	100.00%	WP_264309144.1	
13	+	5871	6035	54	hypothetical protein	Riemerella anatipestifer	96%	2e-23	88.46%	WP_153938400.1	
14	+	6035	6322	95	DNA methyltransferase	Riemerella phage RAP44	100%	4e-60	97.89%	YP_007003654.1	
15	+	6393	7160	255	phosphoadenosine phosphosulfate reductase family protein	Riemerella anatipestifer	100%	0.0	96.47%	WP_260390787.1	
16	+	7173	7619	148	hypothetical protein	Riemerella phage vB_RanS_CRP19	100%	3e-105	97.30%	WIT94480.1	
17	+	7624	7890	88	hypothetical protein	Riemerella phage vB_RanS_CRP19	100%	4e-57	94.32%	WIT94479.1	
18	+	8152	8448	98	nucleoside triphosphate pyrophosphohydrolase	Riemerella phage vB_RanS_CRP19	100%	5e-71	100.00%	WIT94478.1	
19	+	8450	8920	156	hypothetical protein	Riemerella phage vB_RanS_CRP19	100%	7e-114	100.00%	WIT94477.1	
20	+	8987	9370	127	hypothetical protein	Riemerella phage PJA1	100%	1e-85	97.64%	XMN71838.1	
21	+	9327	9866	179	hypothetical protein	Riemerella phage vB_RanS_CRP19	100%	2e-134	99.44%	WIT94475.1	
22	+	9853	10299	148	hypothetical protein	Riemerella phage vB_RanS_CRP19	100%	6e-112	100.00%	WIT94474.1	
23	+	10283	10459	58	hypothetical protein	Riemerella phage vB_RanS_CRP19	100%	3e-34	100.00%	WIT94473.1	
24	+	10437	10841	134	hypothetical protein	Riemerella phage vB_RanS_CRP19	99%	4e-98	99.25%	WIT94472.1	
25	+	10842	11102	86	hypothetical protein	Riemerella anatipestifer	100%	2e-50	96.51%	WP_064968281.1	
26	+	11099	11371	90	hypothetical protein	Riemerella anatipestifer	100%	3e-58	98.89%	WP_214192823.1	
27	+	11461	11790	109	hypothetical protein	Riemerella anatipestifer	100%	1e-73	100.00%	WP_004917903.1	
28	+	12030	12854	274	DNA-damage-inducible protein D	Riemerella phage vB_RanS_CRP19	100%	0.0	100.00%	WIT94468.1	
29	+	13127	13447	106	hypothetical protein	Riemerella anatipestifer	100%	2e-72	99.06%	WP_004917900.1	
30	+	13450	14199	249	phage antirepressor Ant	Riemerella anatipestifer	100%	0.0	100.00%	WP_004917897.1	
31	+	14262	14576	104	SH3 beta-barrel fold-containing protein	Riemerella anatipestifer	100%	4e-70	98.08%	WP_216706677.1	
32	+	14679	15098	139	recombination protein NinG	Riemerella anatipestifer	100%	9e-98	100.00%	WP_064968278.1	
33	+	15104	15433	109	hypothetical protein	Riemerella anatipestifer	100%	2e-72	98.17%	WP_153938386.1	
34	+	15476	15586	36	hypothetical protein	Caudoviricetes sp.	100%	3e-07	75.00%	DAN80535.1	
35	+	15586	16290	234	phosphoadenosine phosphosulfate reductase domain-containing protein	Riemerella anatipestifer	100%	1e-173	100.00%	WP_064968276.1	
36	+	16409	16990	193	DNA methylase	Riemerella anatipestifer	100%	5e-133	99.48%	WP_340307980.1	
37	+	16980	17462	160	terminase	Riemerella anatipestifer	100%	8e-115	100.00%	WP_310486677.1	
38	+	17557	18855	432	terminase large subunit	Riemerella phage vB_RanS_CRP19	100%	0.0	98.38%	WIT94459.1	
39	+	18969	20327	452	portal protein	Riemerella phage RAP44	96%	0.0	97.71%	YP_007003632.1	
40	+	20327	21370	347	head morphogenesis capsid protein	Riemerella phage vB_RanS_CRP5	100%	0.0	97.12%	WP_310486681.1	
41	-	21568	21365	67	hypothetical protein	Riemerella phage RAP44	100%	9e-48	98.51%	YP_007003628.1	
42	-	21699	21550	49	hypothetical protein	Riemerella anatipestifer	100%	1e-24	100.00%	WP_185094095.1	
43	-	22215	21985	76	hypothetical protein	Riemerella anatipestifer	100%	2e-46	94.74%	WP_250206832.1	
44	-	22495	22229	88	hypothetical protein	Riemerella phage vB_RanS_CRP6	100%	3e-62	97.73%	WIL01307.1	
45	+	22628	23284	218	hypothetical protein	Riemerella phage PJO17	100%	1e-158	99.54%	XMN71888.1	
46	+	23288	23659	123	major head protein	Riemerella phage RAP44	100%	1e-84	95.12%	WP_013447240.1	
47	+	23679	24761	360	major capsid protein	Riemerella anatipestifer	100%	0.0	98.89%	WP_264315013.1	
48	+	24763	25086	107	DUF6706 family protein	Riemerella anatipestifer	100%	2e-70	100.00%	WP_014938290.1	
49	+	25080	25403	107	hypothetical protein	Riemerella anatipestifer	100%	1e-73	100.00%	WP_154508377.1	
50	+	25394	25837	147	hypothetical protein	Riemerella phage vB_RanS_PT33	100%	1e-105	96.60%	UVK80383.1	
51	+	25834	26244	136	hypothetical protein	Riemerella anatipestifer	100%	6e-93	97.79%	WP_061710114.1	
52	+	26271	26777	168	hypothetical protein	Riemerella anatipestifer	100%	2e-117	99.40%	WP_310485794.1	
53	+	26857	27318	153	hypothetical protein	Riemerella anatipestifer	100%	2e-104	97.39%	WP_220332328.1	
54	+	27336	27521	61	hypothetical protein	Riemerella anatipestifer	100%	3e-36	100.00%	WP_014938296.1	
55	-	28737	28045	230	Ig-like domain-containing protein	Riemerella anatipestifer	100%	1e-165	99.57%	WP_216706706.1	
56	+	28924	33561	1545	tape measure protein	Riemerella anatipestifer	100%	0.0	94.77%	WP_214246186.1	
57	+	33558	33665	35	hypothetical protein	Phage vB_RanS_PJN03	100%	3e-14	100.00%	UXN80990.1	
58	+	33662	34093	143	hypothetical protein	Riemerella phage PJO17	100%	1e-109	100.00%	XMN71904.1	
59	+	34090	35127	345	galactose-binding protein	Riemerella anatipestifer	100%	0.0	97.67%	WP_340307982.1	
60	+	35112	39062	1316	phage tail protein	Riemerella anatipestifer	100%	0.0	92.71%	WP_340299250.1	
61	+	39083	39406	107	hypothetical protein	Riemerella anatipestifer	96%	9e-62	95.15%	WP_015345342.1	
62	+	39390	39722	110	hypothetical protein	Riemerella anatipestifer	100%	3e-68	91.82%	WP_220332346.1	
63	+	39719	40240	173	holin	Riemerella phage RAP44	100%	7e-129	99.42%	YP_007003602.1	
64	+	40230	40808	192	hypothetical protein	Riemerella phage vB_RanS_CRP6	100%	2e-125	93.75%	WIL01283.1	
65	+	40831	41385	184	N-acetylmuramoyl-L-alanine amidase	Riemerella anatipestifer	100%	6e-132	97.28%	WP_049334505.1	
66	-	41694	41410	94	helix-turn-helix transcriptional regulator	Riemerella anatipestifer	100%	4e-58	100.00%	MFL1601820.1	
67	+	42119	42748	209	hypothetical protein	Riemerella anatipestifer	100%	8e-150	100.00%	MFL1601819.1	
68	+	42879	42968	29	hypothetical protein	Buchnera aphidicola	79%	3.2	50.00%	WP_343188257.1	
69	+	42980	43303	107	hypothetical protein	Riemerella anatipestifer	100%	2e-72	100.00%	MFL1601818.1	
70	+	43328	43576	82	hypothetical protein	Riemerella phage vB_RanS_CRP19	100%	6e-61	100.00%	WIT94502.1	
71	+	43810	44151	113	hypothetical protein	Riemerella anatipestifer	100%	7e-76	97.35%	WP_004917809.1	
72	+	44151	44333	60	DNA-binding protein	Riemerella phage vB_RanS_CRP5	98%	3e-26	74.58%	WCS66409.1	
73	-	45688	44372	438	hypothetical protein	Riemerella phage vB_RanS_CRP19	100%	0.0	99.54%	WIT94499.1	
74	-	46010	45741	89	hypothetical protein	Riemerella anatipestifer	100%	3e-55	96.63%	WP_064968301.1	
75	-	46459	46007	150	 hypothetical protein	Riemerella anatipestifer	100%	1e-105	100.00%	WP_064968300.1	
